# Emerging paradigm of virtual-microscopy for histopathology diagnosis: survey of US and Canadian oral pathology trainees

**DOI:** 10.1038/bdjopen.2017.13

**Published:** 2017-07-28

**Authors:** Ngozi N Nwizu, Adepitan Owosho, Kalu U E Ogbureke

**Affiliations:** 1 Department of Diagnostic and Biomedical Sciences, The University of Texas School of Dentistry at Houston, Houston, TX, USA; 2 Department of Surgery, Dental Services, Memorial Sloan-Kettering Cancer Center, New York, USA

## Abstract

**Objectives/Aims:**

The application of virtual microscopy (VM) to research, pre-doctoral medical and dental educational training, and diagnostic surgical and anatomic pathology is well-documented but its application to the field of oral and maxillofacial pathology has not been explored. This is the first study to evaluate the enthusiasm and readiness of US-/Canada-based oral and maxillofacial pathology (OMFP) residents toward employing VM use over conventional microscopy (CM) for diagnostic purposes.

**Materials and Methods:**

All 46 current US-/Canada-based OMFP residents were invited to participate in an anonymous electronic survey via ‘Survey Monkey’ in 2015. The survey comprised sixteen multiple choice questions and two ‘free text’ questions.

**Results:**

14% of respondents of the 22 (48%) respondents who completed the survey indicated a willingness to substitute CM with VM in <5 years, and 33% within 10 years. 52% reported they would never substitute CM with VM. Approximately 10 and 57% of respondents thought VM will become an acceptable sole diagnostic tool in most centers within 5 and 10 years, respectively. These findings are irrespective of the fact that overall, 90% of respondents reported being familiar with VM use.

**Discussion:**

VM technology is unlikely to substitute CM in diagnostic oral and maxillofacial histopathology practice among future OMFP practitioners in the foreseeable future.

## Introduction

The concept of virtual microscopy (VM) has gained considerable attention within the last two decades. This technology utilizes digitization of whole-microscopic glass slides via computer-aided systems to produce virtual microscopic slides for the interpretation of histology and histopathologic tissue sections. Theoretically, VM should perfectly recapitulate the conventional microscope (CM) with glass slide systems such that histologic sections can be evaluated without loss of quality. VM is now commonly used in research settings^1,2,3^ and has been incorporated into the histology, pathology and oral pathology curricula of several educational institutions for the training of its medical and dental students.^4,5,6,7,8,9,10,11,12,13^ In many of these instructional settings the response to the introduction of VM has been overwhelmingly positive, sometimes resulting in a complete phase out of conventional microscopy (CM).^4,7,9,10^ Commonly adduced reasons for its popularity relative to CM include ease of navigation while maintaining orientation, better or at par image quality, and facilitation of learning through inclusion of digital annotations and legends with additional informational text.^6,13,14^

Other VM ‘likeability’ factors proffered by students include slide consistency across board, greater time efficiency, excellent flexibility including remote access to virtual slides, and increased student interactions.^15,16^ Although initial costs involved in transitioning from CM to VM use can be quite high, however, once the conversion process is complete, those digital images can be preserved and used indefinitely at minimal cost. VM use therefore becomes a more efficient and cost effective process in the long run as it obviates the need to maintain a functioning laboratory, and qualified laboratory staff to continually produce adequate representative tissue sections and maintain high quality histological slides.^5,12,13,14,15,16,17^

The rapid, sustained improvement in the quality and range of VM functionalities over time, however, has resulted in an enhanced expectation beyond instructional and research settings, to include its ultimate applicability to diagnostic histopathology. In certain circles, VM-assisted technologies have broadened significantly to include routine diagnostic surgical pathology services, frozen tissue sections evaluation, remote second opinion consultations/collaborations,^18,19^ and quality assurance exercises.^20,21,22^ In addition, VM has proved a useful adjunct in the training and competency assessments of pathology residents,^21^ virtual continuing education pathology courses^20^ and the American Board of Oral and Maxillofacial Pathology annual quarterly slide review program. Future projections include more widespread usage in competency assessment in the surgical pathology section of Residency In-Service Examination (RISE) and associated board certification examinations.^21,23^

Use of VM has its disadvantages however, including slow image download speeds, time consuming nature of slide digitization, and need for dedicated servers and large data storage space,^10,11,16^ although newer VM software appear to have circumvented some of these problems. Significant concerns among pathologists persist ranging from accuracy of diagnosis, other quality control issues and associated medico-legal implications.^15,24^ This is in spite of some evidence to indicate that diagnostic accuracy of VM is comparable to CM (the ‘gold standard’).^25,26^ These lingering concerns account for cautious optimism and a lack of scientific consensus among pathologists regarding the acceptance of VM as a credible stand-alone tool for diagnostic histopathology.

The impact of virtual microscopy on the pre-doctoral pathology education of dental and medical students, and training of medical residents in surgical and anatomic pathology, has been reported extensively in the scientific literature.^7,8,9,11,12,13,14,27^ However, no study has specifically targeted oral and maxillofacial pathology (OMFP) trainees. The objective of this study therefore was to investigate the enthusiasm and readiness of current U.S. and Canadian-based oral and maxillofacial pathology (OMFP) residents to a potential paradigm of fully replacing CM with VM for diagnostic histopathological purposes.

## Materials and Methods

Appropriate Institutional Review Board Approval from the University of Texas Health Science (UTSD) at Houston was obtained prior to commencement of study. An email listing of all current residents in the United States as at year 2015, obtained from the American Academy of Oral and Maxillofacial Pathology (AAOMP) site, was used to invite all OMFP residents in the United States and Canada to participate in our anonymous electronic survey that same year. A total of three reminders were sent to potential respondents over a six-month period. The online survey utilized a questionnaire format based on the ‘Survey Monkey’ software to capture information on readiness of VM use among OMFP residents in the United States and Canada. Structured self-reported questionnaires have been widely used as a valid tool to evaluate the perceptions and application of virtual microscopy in predoctoral (medical and dental) education, and among anatomic pathology trainees/pathologists.^7,8,9,11,12,13, 14,27^ Our questionnaire was developed in consultation with our institution’s manager of educational technology (RH), well versed in survey designs and educational technology. The survey was pre-tested informally among a few faculty members to ensure the questions were appropriate and captured the information we intended to collect. The data was non-linked in order to protect the confidentiality of the participants and information collected was collated via an excel data collection form. Statistical analysis was primarily descriptive in nature producing frequency counts, means, percentages and graphical representations.

There are eighteen American Dental Association (ADA) accredited OMFP residency programs in the United States (16) and Canada (2), with a total of 46 current residents. Programs range in design from 3-year ‘certificate-only’ to combined certificate with Masters/PhD/other doctorate programs (4–5 or more years). Twenty-two of the 46 residents responded to and completed the survey. A total of eighteen questions were administered to the participants: sixteen multiple choice questions including two Likert-scale type questions on a scale 0–4 (Question 10, 14), and 2 ‘free text’ questions. Participants were instructed to choose only one answer for each of the multiple choice type questions. Questions 1 through 6 and questions 16 and 17 collected information on participants’ demographics, while questions 7 to 9 focused on their intention to sit for the relevant board exams and where to practice OMFP following graduation, respectively. Questions 10–15 captured specific information relative to VM use. These include participants’ perceived knowledge of the concept of VM (Question 10), if their application of VM has been restricted to research purposes only (Question 11), or for didactic learning of histology/pathology (Question 12). Other pertinent VM questions related to whether aspects of their residency training incorporated VM use (Question 13), their perception of the overall popularity of VM use in the short and long term across OMFP centers (Question 14), and their own personal willingness to substitute CM use for VM in the near and distant future (Question 15). Of the two free text questions, the first (Question 9) determined what countries trainees planned to practice oral pathology (United States or other countries) after graduation. The second (Question 18) was designed to gain additional insights into individual trainees’ views on VM use for diagnostic. A copy of the questionnaire can be viewed in the ‘Online Supplementary Information’ section.

## Results

**Figure 1:**
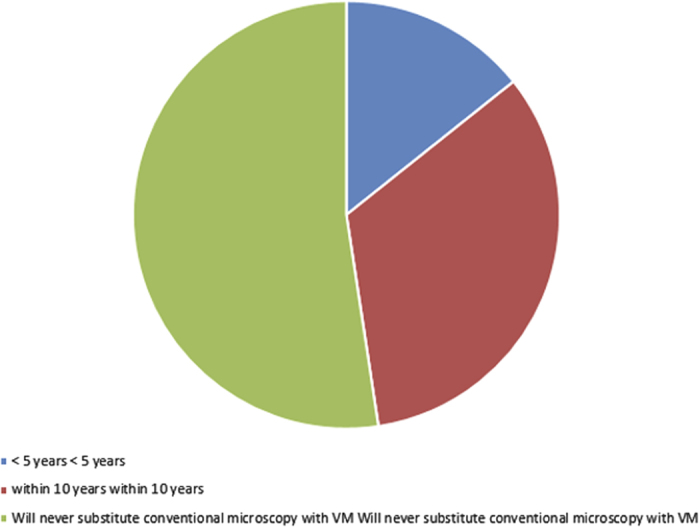
Pie chart illustrating the distribution of response to Q15 of the questionnaire: given your current opinion of the state of the art of VM, how soon do you estimate you will be ready to substitute CM with VM (<5years/within 10years/will never substitute)?.

**Figure 2:**
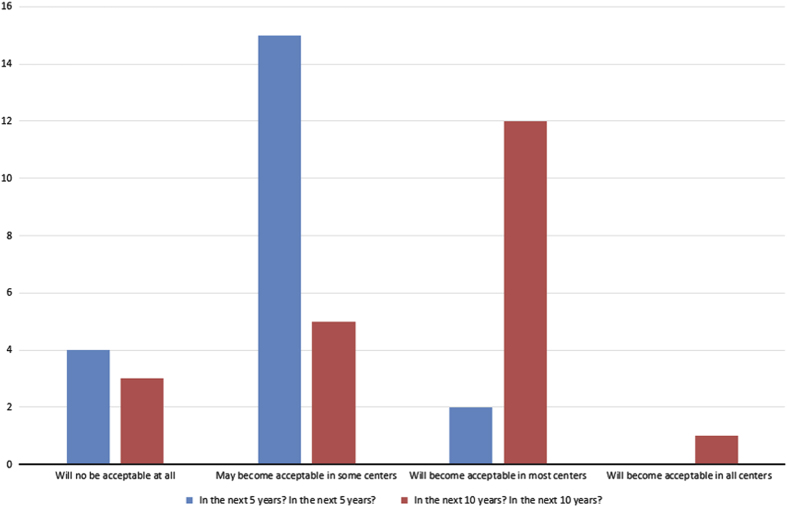
Bar chart illustrating the distribution of response to Q14 of the questionnaire: how will you rate the acceptability of VM as a sole tool for histopathologic diagnosis in the United States (in the next 5/10years)?.

**Figure 3:**
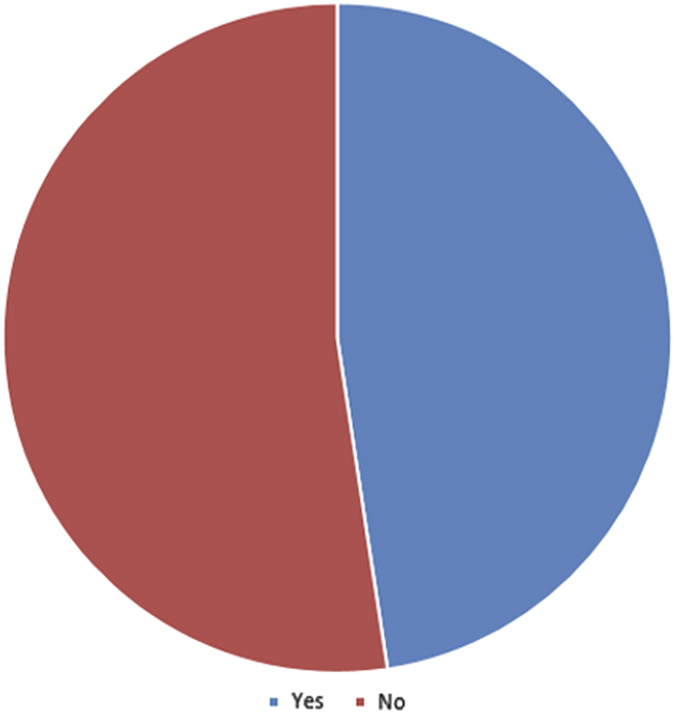
Pie chart illustrating the distribution of response to Q13 of the questionnaire: have you employed received aspects of your residency training instructions through VM?.

**Figure 4:**
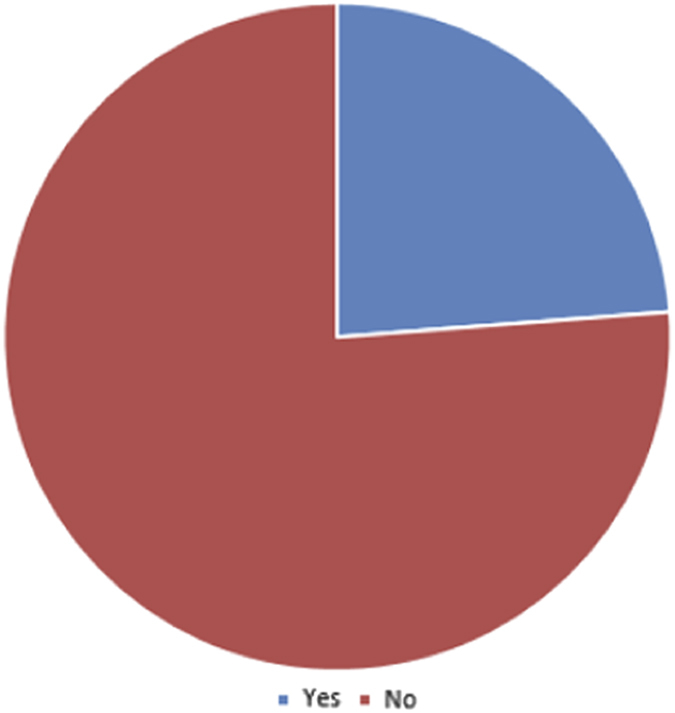
Pie chart illustrating the distribution of response to Q11 of the questionnaire: have you employed VM in the acquisition of purely research data.

**Figure 5:**
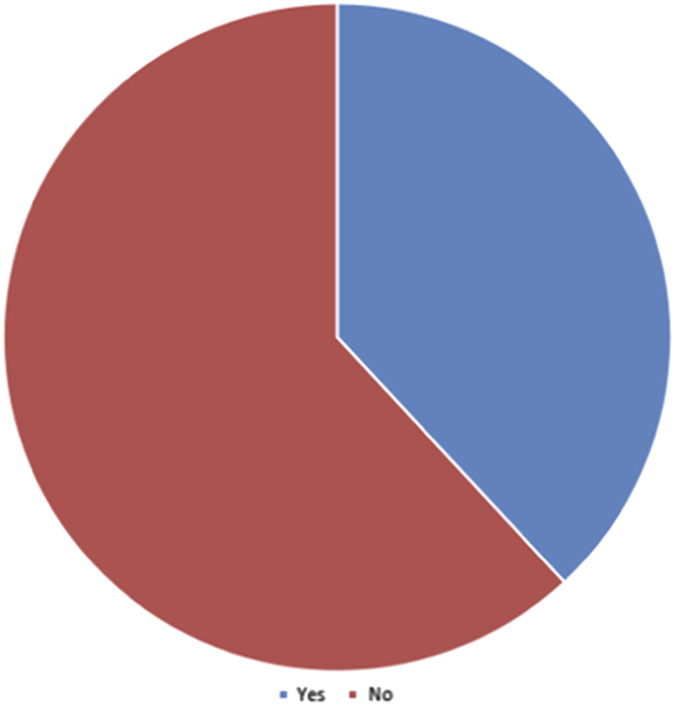
Pie chart illustrating the distribution of response to Q12 of the questionnaire: have you employed VM purely for classroom instruction in histology/pathology?.

**Figure 6:**
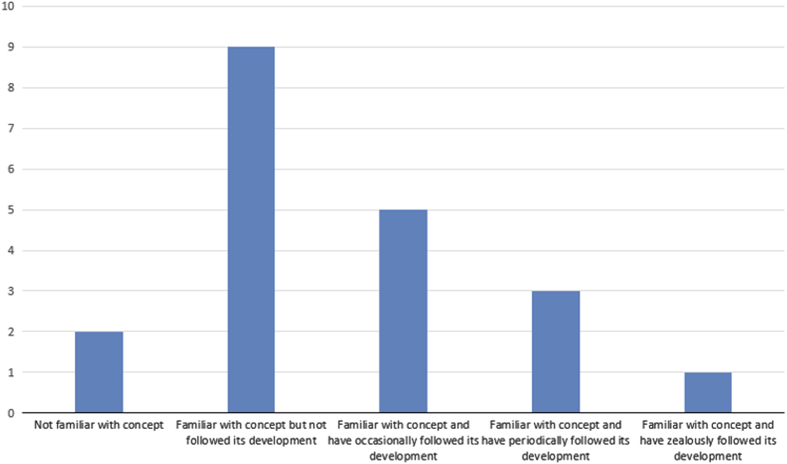
Bar chart illustrating the distribution of response to Q10 of the questionnaire: assess your knowledge of the concept of virtual Microscopy (VM) on a scale of 0 to 3.

The VM-specific questions (Questions 10–15), as described in the Methods section, are summarized in Figures 1–6. Of the 46 residents invited to participate in the survey questionnaire, 22 (~48%) completed the survey. Respondents comprised 11 males (~52%) and 10 females (~48%). Seventeen (~81%) respondents reported possessing excellent written and spoken English language skills, with only one respondent (~5%) indicating a fair command of written English language. The dental degrees earned by respondents were DDS eight (~38%), BDS eight (~38%) and DMD four (~19%); none had a medical degree in addition to a dental degree. Nine (~43%) respondents indicated their programs offer a ‘certificate-only’, while 10 (~48%) and 2 (~10%) indicated their programs offer combined certificate with Master’s and PhD degrees, respectively. In addition to a dental degree, three (~14%) trainees had a doctorate (PhD) degree, one (~5%) a PhD and a Master in Public Health (MPH degrees. None of the respondents had a prior master’s degree.

All of the respondents anticipate completion of residency program between 2015 and 2017: eight (~38%) in 2015; five (~24%) in 2016; and eight (~38%) in 2017 (Question 6). All (100%) planned to challenge the American Board of Oral and Maxillofacial Pathology (ABOMP) certification examinations and attain ‘Diplomate’ status on completion of their residency training (Question 7). Furthermore, 68% of respondents plan to practice oral and Maxillofacial Pathology (OMFP) in the United States, or Canada (Question 8), while 32% plan to practice in other countries: Saudi Arabia (~19%); and Canada (~10%), respectively (Question 9).

Fourteen percent of respondents estimated they will be ready to substitute CM with VM in less than five years, and 33% within the next ten years, but 52% indicate they will never substitute CM with VM (Question 15, Figure 6). Overall, these results indicate that almost half (47%) of respondents will be ready to substitute VM for conventional microscopy by the next decade (Question 15, Figure 6). None of the respondents had any additional comments regarding their position on VM for diagnostic histopathology (Question 18). Most participants (71%) expect VM use may become acceptable in some institutions as the sole microscopic tool for diagnostic histopathology in the United States within the next five years. None of the participants (0)% anticipate VM use would become widely accepted enough to become available at all OMFP centers within that time frame, although 1 participant (5%) felt this was achievable by 10 years (Question 14, Figure 5).

Almost half of study participants (48%) admitted to receiving aspects of their training instructions via VM (Question 13, Figure 4). Whereas 24% indicated they have employed VM use only for the acquisition of research data, (Question 11, Figure 2), 38% reported incorporating VM in classroom teaching of histology/pathology (Question 12, Figure 3). In general, with respect to familiarity with the concept of VM, 10% of respondents indicated non-familiarity, 45% ‘familiar, but not followed its development’, 40% ‘familiar, and have occasionally or periodically followed its development’, and 5% ‘familiar, and have zealously followed its development’ (Question 10, Figure 1).

## Discussion

Virtual microscopy was first introduced into the digital landscape of histopathology almost twenty years ago. Since then the technology has undergone series of evolution prompting its incorporation into the histology and histopathology courses of medical and dental students in many schools^4,5,6,7,8,9,10,11,12,13^ and training of some pathology residents.^21^ VM technology has also been used in some settings to augment CM in the provision of diverse routine diagnostic surgical pathology services,^18,19^ including quality assurance re-reviews.^20,28,29^ Yet, many pathologists remain skeptical toward its full integration into mainstream routine diagnostic histopathology as a credible alternative to conventional microscopy^17,24^ This is reflected in the results of our survey which found that only about 14% of the US/Canadian OMFP trainees polled indicated they would be ready to substitute CM for virtual microscopy in less than five years. It is also instructive that about 52% of the respondents stated they would never substitute conventional microscopy with virtual microscopy. This is despite 90% of respondents admitting to familiarity with use of VM, and integrating VM technology into histology/histopathology courses (~38%), or research (~24%).

These findings are significant because almost 50% of all OMFP residents in the U.S. and Canada participated in our survey and all plan to sit for the American Board of Oral and Maxillofacial Pathology examinations, while 68% will practice in the United States. Our study findings are very similar to those of Bellis *et al.*,^27^ which examined attitudes and practices among pathologists and pathology residents in Canada. They reported that although 90% of respondents were conversant with the use of VM, and 71% believed VM technology was needed in their practice, only a fraction of pathologists (38%) were amenable to the idea of VM use for routine diagnostic histopathology.^27^ In contrast, a dermatopathology in-training examination administered to US dermatology residents showed no particular preference between glass slides and VM.^30^

Another important aspect of our study is that only about 10% of respondents polled thought the substitution of CM for VM in routine diagnostic histopathology will definitely become a common phenomenon across health institutions in the United States within the next 5 years. However, this figure rose significantly to 57% within the next 10 years, suggesting that VM use in routine diagnostic histopathology among US-/Canadian-based OMFP pathologists may increase in popularity, albeit slowly, within the next decade. There appears to be legitimate reasons why many pathologists continue to balk at the concept of endorsing VM use in place of CM in the provision of routine diagnostic surgical pathology services. One important lingering concern is the perception by some practitioners that the image quality and representative tissue sections are not adequately reflected in VM at the moment.^27^ As a consequence, pertinent histologic features may be missed which may lead to an erroneous diagnosis and its attendant medico-legal implications. A study that examined the diagnostic accuracy and acceptability of VM slides of breast needle core biopsies among pathologists, found an agreement between the original glass slide diagnosis and its subsequent examination via VM in nine out of ten slides examined. The concordance rates for slides ranged from 35.3 to 100% with an average concordance rate between slides of 65%. A key finding of their study however, was that only 6.25% of participants reported being ‘very confident’ about their diagnosis, although 18.75% and 56.25%, respectively described their confidence levels as ‘confident’ and ‘reasonably confident’. Paradoxically, 87.5% of the participants were satisfied with the level of image quality with 18.75% indicating they were of excellent quality.^31^ Higher concordance rates have been noted in other studies.^32,33^ Many studies in general found that VM was comparable to CM in diagnostic accuracy,^34,35,36^ and that VM images were of sufficiently good quality to make the correct diagnosis.^26^ Conversely, a large study involving over 1000 cases found major discrepancies in eighteen of those cases, although none of these were associated with potentially serious outcomes such as neoplasia. The areas of discordance were mainly attributable to oversight of small focal findings in the slides examined.^37^

Some other barriers to VM becoming the sole tool in diagnostic histopathology include high costs^38^ particularly for smaller health institutions, lack of standardized guidelines on validation of VM for the diagnostic histopathology purposes,^39^ and unfamiliarity with VM technology. Many pathologists still consider high costs of VM installation and maintenance as a deterrent to adopting VM use in diagnostic histopathology,^38^ although rapid improvement in VM technology over the years has resulted in more affordable and better quality equipment.^40^ Currently, guidelines on validation of VM for diagnostic purposes are at best unclear, thereby increasing the possibility for patient care compromise. The College of American Pathologists Pathology and Laboratory Quality Center recently issued a 12-point set of recommendations intended to guide pathology laboratories in establishing procedures for the validation of their VM systems. Basically, it recommends that a validation study be carried out by pathologists skilled in use of VM systems and should include 60 routine cases each time, with intra-observer concordance rates between digitized and glass slides determined after a minimum period of 2 weeks.^39^

The reasons for unfamiliarity with VM technology may be two pronged. One is the lack of adequate exposure to VM technology. This may be particularly true for seasoned pathologists who, unlike their younger colleagues, may not have been exposed to VM technology during their dental school or residency training. Findings from a study by Brick *et al.*^30^ buttress this argument. The investigators found an overall statistically significant difference in the diagnostic accuracy between VM and CM use among its residents who examined a total of forty-eight slides (*P*=0.01). Interestingly, when the study was restricted to first-year trainees alone or trainees with more than two exposures to VM per month, no such difference was noted.^30^ The investigators concluded that year of residency training coupled with prior experience with VM are contributing factors in its diagnostic accuracy. These first year residents may have been beneficiaries of the increasing use of VM technology across US academic institutions. Similarly, unlike recent graduates who grew up in the digital age and are more likely to be technologically savvy, older pathologists may harbor some dislike for VM technology because of technophobia. In a study by Bellis *et al.*,^27^ more pathologists (13.7%) than residents (10.3%) surveyed expressed their unease with VM use, and some pathologists (2.9%) but no residents considered the amount of time to learn the technology was a barrier to its use. In our study, although 90% of respondents were familiar with the concept of VM, and almost 48% had received some practical training in VM, the vast majority (~86%) indicated a lack of willingness to adopt VM in diagnostic histopathology in the near future (<5 years). This suggests that among future US and Canada-based OMFP pathologists, there are other more pertinent factors barring their use of VM exclusively in diagnostic histopathology besides familiarity with VM use. In spite of these peculiar VM-related challenges, the distinct advantages of VM over CM such as portability, flexibility, convenience, long term cost effectiveness and ease of use for indefinite storage and retrieval of digital images in a secure environment,^7,8,15,17,41^ are likely to ensure its appeal among pathologists will wax rather than wane in the long term.

In presenting our data, the limitations of our study need to be taken into consideration. One obvious limitation is the inherently small number of respondents (*n=22*). However, OMFP is a very small specialty program with <50 residents across the United States and Canada. Second, although we did capture respondents from a number of institutions across the United States and Canada, our response rate of 48% may have introduced some nonresponse bias and not be generalizable to all US and Canadian residents. Other limitations include the fact that our evaluation was based entirely on self-report and did not involve any objective means of assessment such as number of cases examined and length of experience. However, our study is unique in that this is the first study to specifically address VM use in relation to diagnostic oral and maxillofacial pathology (OMFP) in the United States and Canada. Although we had limited power to draw any meaningful statistical conclusions, we have been able to provide important baseline information on level of acceptance of VM in diagnostic histopathology among future OMFP practitioners. This could serve as the basis for a more elaborate study design on a larger scale that would include current OMFP practitioners and also address issues pertaining to perceived barriers to the use of VM.

## Conclusion

In summary, VM technology is unlikely to substitute CM in diagnostic oral and maxillofacial histopathology practice among future OMFP practitioners in the foreseeable future, while VM technology develops further to address current concerns and attain universal acceptability as a total substitute to CM. Its full integration into diagnostic histopathology may also hinge on the ability to effectively bridge the divide between proponents and opponents of VM technology through high quality training on VM functionalities and applications through combined efforts of technical experts and pathologists. Other important considerations include education on its inherent long term economic benefits, and an across-the-board adoption of standardized guidelines on validation of VM with respect to diagnostic histopathology.
